# Rare myeloid sarcoma/acute myeloid leukemia with adrenal mass after allogeneic mobilization peripheral blood stem cell transplantation

**DOI:** 10.7497/j.issn.2095-3941.2013.04.008

**Published:** 2013-12

**Authors:** Ya-Fei Wang, Qian Li, Wen-Gui Xu, Jian-Yu Xiao, Qing-Song Pang, Qing Yang, Yi-Zuo Zhang

**Affiliations:** 1 Department of Hematological Oncology, Tianjin Medical University Cancer Institute and Hospital, National Clinical Research Center of Cancer, Tianjin Key Laboratory of Cancer Prevention and Therapy, Tianjin 300060, China; 2 Department of PET-CT, Tianjin Medical University Cancer Institute and Hospital, National Clinical Research Center of Cancer, Tianjin Key Laboratory of Cancer Prevention and Therapy, Tianjin 300060, China; 3 Department of Radiology, Tianjin Medical University Cancer Institute and Hospital, National Clinical Research Center of Cancer, Tianjin Key Laboratory of Cancer Prevention and Therapy, Tianjin 300060, China; 4 Department of Radiotherapy, Tianjin Medical University Cancer Institute and Hospital, National Clinical Research Center of Cancer, Tianjin Key Laboratory of Cancer Prevention and Therapy, Tianjin 300060, China; 5 Department of Urologic Oncology, Tianjin Medical University Cancer Institute and Hospital, National Clinical Research Center of Cancer, Tianjin Key Laboratory of Cancer Prevention and Therapy, Tianjin 300060, China

**Keywords:** Myeloid sarcoma (MS), acute myeloid leukemia (AML), allogeneic hematopoietic stem cell transplantation, multidisciplinary team (MDT)

## Abstract

Myeloid sarcoma (MS) is a rare hematological neoplasm that develops either *de novo* or concurrently with acute myeloid leukemia (AML). This neoplasm can also be an initial manifestation of relapse in a previously treated AML that is in remission. A 44-year-old male patient was diagnosed with testis MS in a local hospital in August 2010. After one month, bone marrow biopsy and aspiration confirmed the diagnosis of AML. Allogeneic mobilization peripheral blood stem cell transplantation was performed, with the sister of the patient as donor, after complete remission (CR) was achieved by chemotherapy. Five months after treatment, an adrenal mass was detected by positron emission tomography-computed tomography (PET-CT). Radiotherapy was performed for the localized mass after a multidisciplinary team (MDT) discussion. The patient is still alive as of May 2013, with no evidence of recurrent MS or leukemia.

## Introduction

Myeloid sarcoma (MS) is a rare extramedullary manifestation of acute myeloid leukemia (AML), which occurs in 3% to 5% of AML cases. This disease is known by various names, including monocytic sarcoma, extramedullary myeloid cell tumor, myeloblastoma, and chloroma. MS may develop *de novo* or concurrently with AML. It can also be an initial manifestation of relapse in a previously treated AML that is in remission. MS can occur anywhere in the body, but the most common sites include lymph nodes, skin, bones, the central nervous system, and soft tissues^1^^,^^2^.

A 44-year-old male patient was diagnosed with AML which developed from testis MS in our department. The patient was treated by allogeneic mobilization peripheral blood stem cell transplantation (allo-MPBSCT) after rapid hematopoietic reconstitution was performed and complete remission (CR) was achieved. Five months later, a mass was discovered in the left adrenal gland by positron emission tomography-computed tomography (PET-CT). Radiotherapy was performed after a multidisciplinary team (MDT) made a diagnosis, and the benefits and risks of the treatment were discussed with the patient and his family. The patient is still alive as of May 2013, with no evidence of recurrent MS or leukemia.

## Case report

A 44-year-old male patient was admitted to the department of hematology. A palpable mass that enlarged rapidly in the past two months was observed in his right testis. Biopsy was performed in a local hospital, and the pathological diagnosis was MS. Bone marrow aspiration revealed 24.5% blast cell infiltration, and flow cytometry indicated that the cells were positive for CD117, CD34, HLA-DR, CD13, CD33, CD15, and myeloperoxidase (MPO), and partly positive for CD38. Cytogenetic analysis showed 48, XY, +8, +13[3]/47, and +8[12]. Molecular genetics analysis showed that FMS-like tyrosine kinase 3-internal tandem duplication (FLT3-ITD), c-KIT, and nucleophosmin (NPM1) gene mutations were negative. A lumbar puncture was performed and biochemical analysis of the cerebrospinal fluid did not reveal myeloid tumor cells. The final histological diagnosis was AML-M2a [French American British (FAB) classification] or unspecified AML [World Health Organization (WHO) classification] based on the aforementioned pieces of evidence^3^.

Systemic chemotherapy was performed after diagnosis. The patient received remission induction therapy (cytarabine at 100 mg/m^2^/day for 7 d and idarubicin at 12 mg/m^2^/day for 3 d), followed by consolidation therapy (high-dose cytarabine at 3 g/m^2^/day for 3 d). Leptomeningeal involvement is less frequent (<3%) in patients with AML than in patients with acute lymphoid leukemia. The right testis of the patient with MS was involved in the current study. Prophylaxis lumbar puncture and intrathecal chemotherapy with cytarabine at 50 mg/day was performed 6 times during induction and consolidation therapies. The patient eventually received allo-MPBSCT, with his sister as donor. After hematopoietic reconstitution, a complete medical examination confirmed that the patient was in prolonged remission.

Follow-up visits were conducted every three months, during which the patient was admitted to our department. In July 2011, the PET-CT detection showed fluorodeoxyglucose (FDG) uptake in the right adrenal gland with a maximal standardized uptake value (SUV) of 6.5. Peripheral blood smear analysis revealed no circulating blast, and bone marrow aspiration showed no abnormal cell morphology. The percentages of blast cells, promyelocytes, and granulocytes were within normal limits. Karyotype analysis of the bone marrow showed a normal 46 XX female complement, and short tandem repeat examination by polymerase chain reaction (PCR) confirmed full donor chimera.

**Figure 1:**
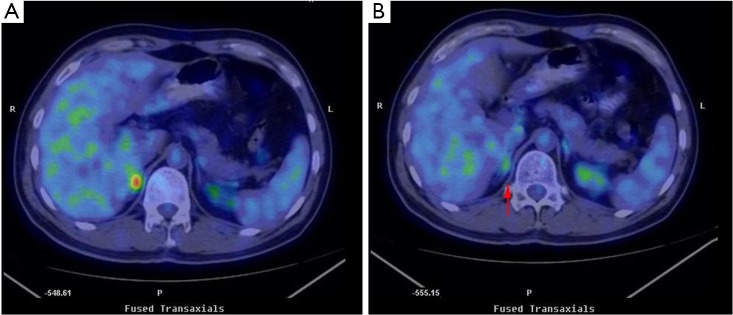
FDG PET-CT results. A, FDG uptake in the right adrenal gland before irradiation. B, FDG uptake disappeared in the right adrenal gland (arrow) after irradiation.

The patient received cyber knife radiotherapy with 1,200 cGy after MDT diagnosis. And benefits and risks of such treatment were discussed with the patient and his family. Repeated PET-CT results showed that FDG uptake disappeared in the right adrenal gland after radiation treatment (**Figure 1**). To date, the patient remains free of symptoms and exhibits no evidence of recurrent MS or leukemia.

## Discussion

MS can occur in any site in the body, and the testicles are among the common sites for this neoplasm. With a mean interval of ten months, MS commonly develops within the first year preceding the occurrence of AML, concomitant with AML, or during AML relapse^1^^,^^2^. The patient in the current study exhibited MS with testicular involvement and was diagnosed with AML one month later.

Bone marrow aspiration and flow cytometry analysis confirmed the diagnosis of AML-M2a (FAB classification). Over the past three decades, AML classification has evolved from the FAB system to the WHO system, which incorporates cytogenetics and dysplasia evidence to refine prognostic subgroups that can define treatment strategies. Molecular genetics analysis of FLT3-ITD, c-KIT, and NPM1 genes showed that the patient had unspecified, intermediate-risk AML (WHO classification)^3^.

AML is a heterogeneous and hematologic malignancy. Treatment of AML is divided into induction chemotherapy and post-remission (or consolidation) therapy based on the National Comprehensive Cancer Network guidelines^4^. Induction therapy of AML patients younger than 60 years old is based on cytarabine and an anthracycline. Idarubicin has longer intracellular retention time than daunorubicin. Consolidation strategies for AML include one or more cycles of high-dose cytarabine followed by allogeneic hematopoietic stem cell transplantation from a matched sibling or an unrelated donor. The patient in the current study received CR induction therapy with cytarabine and idarubicin, followed by consolidation therapy with high-dose cytarabine. The patient underwent allo-MPBSCT, with his sister as donor. Complete medical examination confirmed full donor chimera and that the patient was in prolonged remission.

Adrenal masses are among the most prevalent human tumors. Their overall prevalence ranges from 3% to 10%. Adrenal mass incidence increases with age. The prevalence at autopsy is <1% in patients younger than 30 years old, and it increases to 7% in patients of 70 years old or older. The most common etiologies are as follows: nonfunctioning adenoma (73.9%), subclinical Cushing’s syndrome (7%), aldosterone-producing adenoma (1%), phaeochromocytoma (4.7%), adrenocortical carcinoma (4.8%), and metastases (2.3%)^5^. Malignant masses should be differentiated from benign masses because metastases to the adrenal glands are common^6^. Adrenal gland magnetic resonance imaging (MRI) is often used as a diagnostic method. Normal adrenal glands are hypointense (dark) on both T1-weigthed and T2-weighted images on MRI compared with most soft tissues^7^^,^^8^. PET-CT is an effective method for detecting adrenal masses, and is superior to or at least equivalent to CT or MRI. In addition, a study showed that FDG PET-CT is more precise in detecting lesions than CT or FDG PET alone. First, PET-CT enables early identification of a disease prior to detection by conventional imaging modalities. Second, PET-CT helps in the staging of systemic illnesses. Finally, PET-CT is useful for assessing treatment response after chemotherapy or localized radiotherapy^9^^,^^10^. For the above reported case, through a discussion with the doctors from the Department of PET-CT and Radiology, we agreed that only two possibilities existed, namely, primary tumors and adrenal MS, when the maximal SUV was 6.5. Extramedullary recurrence is rare among AML patients who received bone marrow transplantation. Only 20 (0.65%) cases of extramedullary recurrence among 3,071 AML patients who received transplantation have been reported^11^. We had a discussion with the doctors from the Department of Urologic Oncology to confirm the diagnosis. Biopsy is currently restricted to two indications: obtaining tissue diagnosis prior to initiating treatment in a patient with adrenal metastases on imaging, and less commonly, providing a definitive diagnosis on rare occasions of inconclusive imaging. A timely diagnosis is critical in the latter. Biopsy should not be attempted when an adrenal mass is suspected as a pheochromocytoma because of the potential risk of triggering a hypertensive crisis^7^. The immune functions of a patient is obviously suppressed after transplantation, thus, we believe that biopsy is not the best choice. In the current case, extramedullary recurrence was speculated after a comprehensive analysis of the medical history and radiological changes of the patient.

MS is an unusual disease, thus, defining its outcomes and treatment response is limited. No established guideline for clinical decision making is yet available. Therapies typically include local radiation, systemic chemotherapy, immunotherapy, and donor lymphocyte infusion^1^^,^^2^. Yagi *et al.*^12^ reported the case of a 38-year-old male patient with duodenal MS who underwent allogeneic bone marrow transplantation and additional radiotherapy and achieved CR, which maintained for 21 months. In the current study, the last treatment performed on the patient was cyber knife radiotherapy with 1,200 cGy. The PET-CT result showed that FDG uptake disappeared in the right adrenal gland after irradiation. As of May 2013, the patient remains free of symptoms and exhibits no evidence of recurrent MS or leukemia.

## Conclusion

Extramedullary recurrence is rare among MS/AML patients who received stem cell transplantation. The outcome of the experimental radiotherapy using a cyberknife was approved in the previous diagnosis. No established guideline for clinical decision making in treating extramedullary relapse after allogeneic stem cell transplantation is yet available because of insufficient data. Many patients are already heavily pretreated and may not be able to tolerate chemotherapy, thus, local radiotherapy may be the optimum choice for these patients.

## References

[1] Klco JM Welch JS Nguyen TT Hurley MY Kreisel FH Hassan A State of the art in myeloid sarcoma. Int J Lab Hematol 2011;33:555-565 21883967 10.1111/j.1751-553X.2011.01361.x

[2] Pileri SA Ascani S Cox MC Campidelli C Bacci F Piccioli M Myeloid sarcoma: clinico-pathologic, phenotypic and cytogenetic analysis of 92 adult patients. Leukemia 2007;21:340-350 17170724 10.1038/sj.leu.2404491

[3] The International Agency for Research on Cancer eds. (2008) WHO Classification of Tumours of Haematopoietic and Lymphoid Tissue. IARC Press, Lyon, 2008;140-141.

[4] NCCN Clinical Practice Guidelines in Oncology: Acute Myeloid Leukemia, Version 2. 2012.

[5] Low G Dhliwayo H Lomas DJ Adrenal neoplasms. Clin Radiol 2012;67:988-1000 22486993 10.1016/j.crad.2012.02.005

[6] Low G Sahi K. Clinical and imaging overview of functional adrenal neoplasms. Int J Urol 2012;19:697-708 22462796 10.1111/j.1442-2042.2012.03014.x

[7] Goenka AH Shah SN Remer EM Berber E Adrenal imaging: a primer for oncosurgeons. J Surg Oncol 2012;106:543-548 22886706 10.1002/jso.23235

[8] Taffel M Haji-Momenian S Nikolaidis P Miller FH Adrenal imaging: a comprehensive review. Radiol Clin North Am 2012;50:219-243 22498440 10.1016/j.rcl.2012.02.009

[9] Lee EY Anthony MP Leung AY Loong F Khong PL Utility of FDG PET/CT in the assessment of myeloid sarcoma. AJR Am J Roentgenol 2012;198:1175-1179 22528910 10.2214/AJR.11.7743

[10] Ueda K Ichikawa M Takahashi M Momose T Ohtomo K Kurokawa M. FDG-PET is effective in the detection of granulocytic sarcoma in patients with myeloid malignancy. Leuk Res 2010;34:1239-1241 20493526 10.1016/j.leukres.2010.04.017

[11] Clark WB Strickland SA Barrett AJ Savani BN Extramedullary relapses after allogeneic stem cell transplantation for acute myeloid leukemia and myelodysplastic syndrome. Haematologica 2010;95:860-863 20513805 10.3324/haematol.2010.025890 PMC2878780

[12] Yagi T Ishikawa J Takahashi M Yamashita Y Kusakabe S Yoshinami T Successful Treatment of Duodenal Myeloid Sarcoma with Allogeneic Bone Marrow Transplantation and Additional Radiotherapy. Intern Med 2012;51:769-772 22466836 10.2169/internalmedicine.51.6652

